# The opportunistic protist, *Giardia intestinalis*, occurs in gut-healthy humans in a high-income country

**DOI:** 10.1080/22221751.2023.2270077

**Published:** 2023-10-10

**Authors:** Kristýna Brožová, Milan Jirků, Zuzana Lhotská, Dana Květoňová, Oldřiška Kadlecová, Christen Rune Stensvold, Peter Samaš, Klára J. Petrželková, Kateřina Jirků

**Affiliations:** a Institute of Parasitology, Biology Centre, Czech Academy of Sciences, České Budějovice, Czech Republic; b Department of Medical Biology, Faculty of Science, University of South Bohemia, České Budějovice, Czech Republic; c Institute of Vertebrate Biology, Czech Academy of Sciences, Brno, Czech Republic; d Department of Bacteria, Parasites and Fungi, Statens Serum Institut, Copenhagen, Denmark

**Keywords:** *Giardia intestinalis;* human volunteers, qPCR, conventional-PCR, quantification, contact with animals

## Abstract

*Giardia intestinalis*, a cosmopolitan gastrointestinal protist, is detected mainly in patients with clinical giardiasis in high-income countries. In contrast, there is very little information on the presence of *Giardia* in asymptomatic individuals. Therefore, the aim of this study was to determine the presence and prevalence of *Giardia* in gut-healthy volunteers in the Czech Republic and to perform a comparative evaluation of different diagnostic methods, since *Giardia* diagnostics is complicated. Our results confirmed that the qPCR method is the most sensitive method for detecting *Giardia* and revealed a prevalence of 7% (22/296) in asymptomatic individuals. In most cases, the colonization intensity ranged from 10^−1^–10^1^. A conventional PCR protocol targeting the TPI gene was used to identify the assemblages. However, this protocol had limited sensitivity for *Giardia* amplification, effectively detecting colonization above an intensity of 10^4^. In addition, *Giardia* was detected in 19% of the animals, which were closely associated with the study participants. However, due to methodological limitations, zoonotic transmission could not be clearly confirmed. Notably, contact with animals proved to be the only factor that had a significant impact on the incidence of *Giardia* in gut-healthy humans.

## Introduction

*Giardia intestinalis* is a globally distributed intestinal protist responsible for approximately 180 million symptomatic infections annually and often leading to condition called giardiasis [1, 2]. Infection occurs when individual ingest cysts from contaminated water or food [1]. Nevertheless, several studies have indicated that *Giardia* is frequently present in the stool of asymptomatic children [3–9].

*Giardia* is a complex of organisms exhibit a high degree of genetic diversity and colonizes the small intestine of humans and other mammals [2]. Eight distinct assemblages, referred to as assemblages A-H, have been documented [2]. While assemblages A and B have been found mainly in humans, they display broad host specificity, posing a zoonotic potential [10]. The remaining six assemblages (C–H) are restricted to non-human hosts [11], although sporadic detections in humans have also been reported [12, 13]. Currently, it is considered that *Giardia* assemblages represent distinct species [14].

Various methodological approaches have been employed to detect *G. intestinalis* [15]. Commonly used diagnostic methods still include coproscopic techniques (e.g. flotation or sedimentation), but their predictive value may be affected by the intermittent shedding of cysts in the host excrements and the expertise of the diagnostician [15]. Molecular methods, such as conventional PCR (cPCR) and real-time PCR (qPCR) [15, 16], are also used for detection and assemblage identification. However, the identification of assemblages is impeded by the limited sensitivity of existing molecular genotyping tools, particularly in individuals with weak colonization [17]. Consequently, there exists a knowledge gap regarding the occurrence of assemblages in such samples, and the assessment of zoonotic potential often remains inconclusive [12, 18].

The prevalence of *G. intestinalis* in high-income countries is reported to be between 2% and 7% in high-income countries, while in low-income countries the prevalence is higher and usually ranges from 20% to 30% [19]. Because *Giardia* diagnostic efforts typically target clinical giardiasis cases based on characteristic symptoms, the occurrence of asymptomatic *Giardia* colonization in healthy individuals in high-income countries remains understudied [20]. With increasing interest in gut microbiome research, it has been recognized that asymptomatic *Giardia* colonization is quite common within the human population [3–9]. It is important to note that the available studies on asymptomatic individuals have focused on children under the age of 16 [5, 8]. Here, we sought to address this knowledge by extending our investigation to include a more diverse age range of healthy individuals, from infants to individuals over the age 60.

Given the urgent need to advance our understanding of *G. intestinalis*, its epidemiology, and public health implications pertaining to asymptomatic colonization in the human population, our study aimed to determine the prevalence of this protist among gut-healthy individuals in the Czech Republic (i.e. those without intestinal disease). We further assessed the fecal *Giardia* load in positive samples using qPCR and identified assamblages in selected isolates. Furthermore, we sought to investigate the potential zoonotic transmission of *Giardia* between study participants and their companion animals. To achieve our research objectives, we employed two different diagnostic methodologies such as qPCR targeting SSU RNA, and cPCR assays focusing on three genes, specifically triosephosphate isomerase, beta-giardin, and small ribosomal subunit. One of the sub-objectives was to evaluate and compare the specificity and sensitivity of the above-mentioned cPCR protocols to identify the most robust approach. Finally, we conducted a sensitivity comparison between cPCR and qPCR for the detection of *Giardia*.

## Material and methods

### Sample collection

This study employed samples obtained from our previous investigation focused on another intestinal protist, *Blastocystis* sp [21]. Stool samples origin from volunteers who met the criteria of being gut-healthy (i.e. without diarrhoea, abdominal pain, flatulence, etc.). Samples were collected between 2017 and 2019 in the Czech Republic. The participants also completed a questionnaire providing details regarding gender, age, living location, travelling, animal contact [21]. Additionally, fecal samples were also collected from the animals closely associated with volunteers (for more details see Lhotská *et al*. [21]).

### DNA isolation and PCR diagnostics

Total genomic DNA was extracted from fecal samples (200 mg) using the commercial kit PSP Spin Stool DNA Kit (Stratec, Germany), according to the manufacturer’s protocol (see Lhotská *et al*. [21]).

Initially, we assessed three distinct cPCR protocols, which targeted different genes – specifically, triosephosphate isomerase (TPI), beta-giardin (BG), and the small ribosomal subunit (SSU rRNA) – for their effectiveness in *Giardia* detection. Detailed information on the primers and PCR conditions is provided in Table 1. Each PCR reaction (20 µl) comprised 2×concentrated Master Mix AccuPower® Taq PCR PreMix (Bioneer, Republic of Korea) and was conducted using the T100^TM^ Thermal Cycler (Hercules, California, USA). Positive control DNA was obtained from a trophozoite culture (WB ATCC 30957, human isolate). Visualization of cPCR products (15 µl) was achieved using an electrophoresis system (Thermo Fisher Scientific Inc., USA) with 1.5% agarose gel containing ethidium bromide (0.002 mg/ml). Amplicons were purified using the GenElute^TM^ Gel Extraction Kit (Sigma-Aldrich, MO, USA) and sequencing was outsourced to Eurofins GATC Biotech (Germany).

**Table 1.** Summary of the protocols with specific primers and probe used for conventional PCR and real-time PCR. (SSU-small ribosomal subunit, BG-beta-giardin, TPI-triosephosphate isomerase, F-forward, R-reverse) *-qPCR protocol.

Gene	Lenght of fragment	Primers (5 ´- 3 ´)	PCR conditions	Reference
SSU	292 bp	RH11: CATCCGGTCGATCCTGCC/F	95 °C/3 min, 35× (94 °C/20 s, 63 °C/30 s, 72 °C/1 min), 72°C /5 min	Hopkins et al. [31]
		RH4: AGTCGAACCCTGATTCTCCGCCAGG/R		
BG (nested)	511 bp	G7: AAGCCCGACGACCTCACCCGCAGTGC/F	94 °C/3 min, 35× (94 °C/20 s, 65 °C/30 s, 72 °C/1 min), 72°C /5 min	Cacciò et al. [32]
		G759: GAGGCCGCCCTGGATCTTCGAGACGAC/R		
		BGf: GAACGAACGAGATCGAGGTCCG/F		
		BGr: CTCGACGAGCTTCGTGTT/R		
TPI (nested)	530 bp	G1F: AAATIATGCCTGCTCGTCG/F	95 °C/3 min, 30× (94 °C/45 s, 63 °C/45 s, 72 °C/1 min), 72°C /5 min	Sulaiman et al. [33]
		G1R: CAAACCTTITCCGCAAACC/R		
		G2F: CCCTTCATCGGIGGTAACTT/F		
		G2R: GTGGCCACCACICCCGTGCC/R		
SSU*	62 bp	Giardia 80F: GACGGCTCAGGACAACGGTT/F Giardia 127R: TTGCCAGCGGTGTCCG/R Giardia 105T: FAM-CCC GCG GCG GTC CCT GCT AG-BHQ-1/Taqman probe	10 min at 95 °C followed by 50 cycles of 15 s at 95 °C, 30 s at 60 °C, and 30 s at 72 °C	Verweij et al. [22]

We also applied the qPCR diagnostic protocol [22] utilizing specific primers and a Taqman probe (for details see Table 1). This protocol was optimized for a LightCycler LC 480 I (Roche, Basel, Switzerland) and adapted to our conditions (Master Mix 5x HOT FIREPol® Probe qPCR Mix Plus ROX, Solis BioDyne, Estonia). Each sample was subjected to triplicate analysis (20 µl per qPCR reaction) in a 96-well block, with the positive control identical to that used in cPCR. To estimate the fecal *Giardia* load via qPCR, we established a quantification curve using trophozoite cultures (further details available in Supplementary Data 1).

### Testing DNA inhibition for qPCR

We examined all negative samples for potential qPCR inhibition. This was achieved by adding foreign DNA (from experimental rat tissue) and employing a specific qPCR protocol (commercial primers and Taqman probe for detection of the rat beta-2-microglobulin; ThermoFisher Scientific, Waltham, MA, USA) according to study Šloufová *et al*. [21]).

### Flotation

All fecal samples were also examined coproscopically, using modified Sheather flotation (specific gravity 1.33) [23].

### Statistical analyses

We conducted the McNemar test with Yates correction (0.5) to evaluate the difference in sensitivity between two molecular techniques (qPCR and cPCR) for *Giardia* detection. The calculations were performed using SciStatCalc 2013 software.

(https://scistatcalc.blogspot.com/2013/11/mcnemars-test-calculator.html).

We employed a Bayesian generalized linear model with a binomial distribution and logit link function to investigate the impact of several variables on *Giardia* occurrence as detected by qPCR. The variables considered were age (continuous; years), gender (binary; male/female), living location (binary; city/village), travelling (categorical; non-travel/Europe/outside Europe), and contact with animals (categorical; no contact/contact with pets/contact with pets and livestock) (for more details, see Supplementary data 2).

## Results

### Prevalence of *Giardia intestinalis*

In this study, we examine a total of 431 fecal samples, consisting of 296 samples from asymptomatic humans and 135 animal samples collected within 14 regions of the Czech Republic (for more details, see Lhotská *et al*. [21]).

Initially, we utilized cPCR targeting the TPI gene (530 bp) to analyze *Giardia*-positive samples, as it proved to be the most efficient protocol when compared to alternative protocols for BG and SSU rRNA. The latter two protocols exhibited low specificity and sensitivity (see Supplementary Data 3 for details).

Due to its low sensitivity observed during optimization, we employed the TPI protocol to analyze only a subset of the sample set. This low sensitivity was determined through experiments with *G. intestinalis* culture dilutions ranging from 10^−1^–10^4^ (Supplementary Data 4). Consequently, we restricted our TPI protocol testing to 142 out of 431 samples (101 from humans and 41 from animals). Of these, only seven samples were positive, exhibiting qPCR Ct values between 24 and 28 (Table 2). Specifically, *Giardia* was detected in five rabbits (assemblage BIV; samples no. B151/18, B171/18, B174/18, B263/18, B315/18), one dog (assemblage AII; sample no. B241/18), and one human (assemblage BII; sample no. B441/20). The sequences for these findings are accessible under GenBank numbers OR085318-OR085322 and OR270937-OR270938.

**Table 2.** Summary of qPCR-positive human and animal samples (N = 47) for *Giardia intestinalis* with Ct values and comparison with cPCR results.

Sample	Methods				Sample	Methods			
	cPCR	qPCR	Ct value	Host		cPCR	qPCR	Ct value	Host
B441/20	+	+	25	human	B174/18	+	+	25	rabbit
B6/14	-	+	30	human	B263/18	+	+	25	rabbit
B16/14	-	+	30	human	B171/18	+	+	28	rabbit
B25/14	-	+	30	human	B315/18	+	+	28	rabbit
B227/18	-	+	33	human	B217/18	-	+	34	goat
B246/18	-	+	34	human	B243/18	-	+	35	dog
B233/18	-	+	35	human	B258/18	-	+	35	dog
B60/15	-	+	35	human	B206/18	-	+	35	pig
B77/16	-	+	35	human	B264/18	-	+	35	guinea pig
B231/18	-	+	36	human	B142/17	-	+	36	pig
B248/18	-	+	36	human	B230/18	-	+	36	dog
B436/19	-	+	36	human	B127/18	-	+	37	horse
B9/18	-	+	37	human	B141/18	-	+	37	cat
B87/17	-	+	37	human	B273/18	-	+	37	dog
B121/18	-	+	37	human	B295/18	-	+	37	cat
B147/18	-	+	37	human	B133/18	-	+	38	dog
B429/19	-	+	37	human	B139/18	-	+	38	dog
B442/20	-	+	37	human	B159/18	-	+	38	dog
B123/18	-	+	38	human	B242/18	-	+	38	cat
B255/18	-	+	38	human	B216/18	-	+	38	cow
B64/16	-	+	45	human	B169/18	-	+	39	dog
B66/16	-	+	45	human	B221/18	-	+	39	cat
B151/18	+	+	24	rabbit	B137/18	-	+	40	dog
B241/18	+	+	25	dog					

Following the results from cPCR, all 431 samples were subjected to more sensitive diagnostic qPCR protocol, which revealed a prevalence of 7% (22/296) in asymptomatic humans and 19% in animal samples (25/135) (Table 3). *Giardia intestinalis* was detected in various animal samples including dogs (10/54), cats (4/19), rabbits (5/13), pigs (2/3), cows (1/2), goats (1/4), guinea pigs (1/2), and horses (1/15) (Table 3). In one case, *Giardia* was detected in both an animal (rabbit; sample no. B151/18; Table 2) and its the owner`s samples (sample no. B147/18). However, due to the limited sensitivity of the TPI protocol, we were unable to obtain the sequence from the owner`s sample to determine assemblage (the intensity of colonization < 10^4^). So, the zoonotic potential could not be evaluated.

**Table 3.** List of human and animal species included in this study subjected to qPCR.

Host	Category	N	N positive
*Homo sapiens*	human	296	22 (7%)
*Canis lupus familiaris*	dog	54	10 (19%)
*Oryctolagus cuniculus domesticus*	rabbit	13	5 (39%)
*Felis silvestris catus*	cat	19	4 (21%)
*Sus scrofa domestica*	pig	3	2 (67%)
*Cavia aperea porcellus*	guinea pig	2	1 (50%)
*Equus caballus*	horse	15	1 (7%)
*Bos primigenius taurus*	cow	2	1 (50%)
*Capra aegagrus hircus*	goat	4	1 (25%)
*Anas platyrhynchos domesticus*	duck	1	0
*Anser anser domesticus*	goose	1	0
*Gallus gallus domesticus*	poultry	8	0
*Columba livia domestica*	pigeon	1	0
*Psittacus erithacus*	parrot	1	0
*Ovis aries*	sheep	6	0
*Phodopus sungorus*	hamster	1	0
*Atelerix albiventris*	hedgehog	1	0
*Testudo horsfieldii*	turtle	2	0
*Phelsuma madagascariensis*	felsuma	1	0

Additionally, we verified the presence of *Giardia* in the qPCR amplicons using Sanger sequencing. However, it is important to emphasize that this sequencing did not provide information about specific assemblages. This limitation arises from the conservative nature of the SSU rRNA gene and the very short length of the amplicon (62 bp).

For all 47 positive samples, we identified the fecal *Giardia* load based on the correlations between obtained Ct values and quantification (10^−1^-10^5^ cells per qPCR reaction; Table 2 and Table 4; Supplementary data 1). In most cases (81%), the fecal load corresponded to values of 10^−1^-10^1^ cells per one qPCR reaction.

**Table 4.** Evaluation of the fecal load of *Giardia intestinalis* in human samples based on the quantification curve (set in the range of 10^−1–^10^5^ trophozoites per 1 qPCR reaction).

Estimated fecal Giardia load	Number of samples/ Number of positive samples	Ct value range
10^1^-10^−1^	38/47	30–45
10^2^	2/47	28–30
10^4^	6/47	25–28
10^6^	1/47	24

Internal inhibition was not detected in any of the samples. Additionally, all 431 samples underwent testing using Sheather's flotation method, and only two samples tested positive (human sample no. B441/20 and rabbit sample no. B151/18). Interestingly, these samples exhibited higher *Giardia* loads, specifically 10^4^ and 10^6^.

### Sensitivity comparison of molecular diagnostics methods

To evaluate the difference in sensitivity between cPCR and qPCR, we compared the 142 samples that were tested by both methods. While cPCR detected *G. intestinalis* in only seven cases (7/142), qPCR detected *Giardia* in 24 cases (24/142) (Table 5), indicating that qPCR is the more sensitive method for detecting *Giardia* in fecal samples (McNemar test, χ^2 ^= 18.01; *p* < 0.01; Table 5).

**Table 5.** Comparison of the results of two diagnostic methods (cPCR and qPCR) in detection of *Giardia intestinalis* in 142 samples using McNemar's test (χ^2 ^= 18.01; *p* < 0.01).

		**qPCR**		
		positive	negative	
**cPCR**	positive	7	0	7 (4%)
	negative	17	118	135 (96%)
		24 (17%)	118 (83%)	142

### Factors influencing *Giardia intestinalis* occurrence

**Figure 1.**
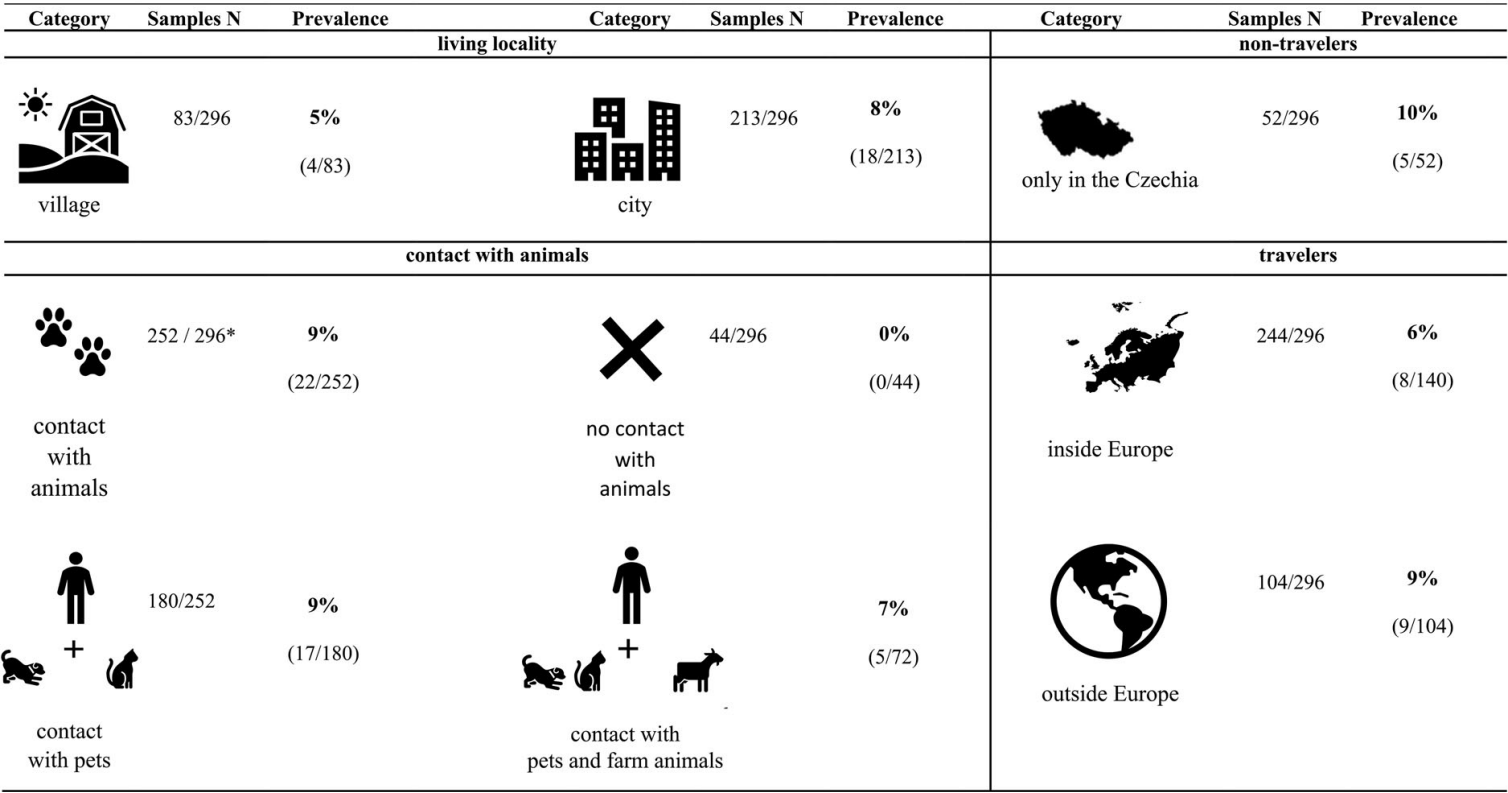
Prevalence of *Giardia intestinalis* in human samples according to the specific categories such as lifestyle (village life/city life, travelling) and contact with animals (pets and farm animals). (Samples N – number of samples obtained in each category out of the total number of samples) * Statistically significant differences.

The impact of specific factors (Figure 1) on *G. intestinalis* prevalence was exclusively investigated within a subset of human samples (N = 296). Supplementary Data 5 presents information concerning asymptomatic *Giardia*-positive individuals.

The incidence of *Giardia* did not exhibit significant differences between rural (5%; 4/83) and urban (8%; 18/213) populations. Concerning travel history, the incidence of *Giardia* was 10% among non-travellers (5/52), 6% among European travellers (8/140), and 9% among individuals who also travelled outside of Europe (9/104).

**Figure 2.**
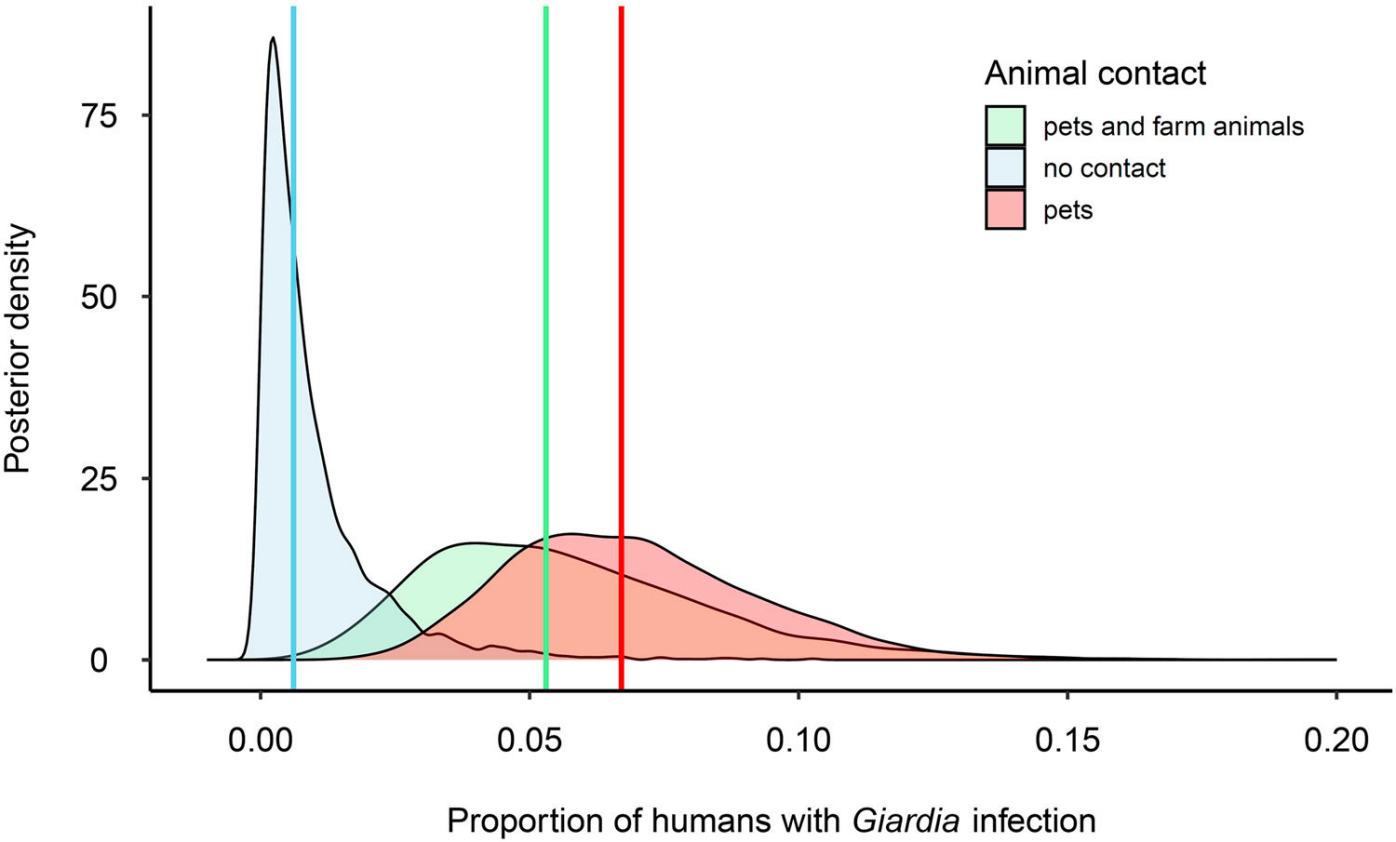
Effect of contact with animals on the probability of *Giardia* infection in humans. We present the entire posterior distribution of the three animal contact categories with their median (vertical line) generated from posterior draws of a Bayesian model.

A higher incidence of *Giardia* was observed in individuals with animal contact (9%; 22/252) compared to those without any animal contact (0/44). Notably, the type of animal (domestic or farm) did not appear to influence *Giardia* prevalence. The outputs from a Bayesian generalized linear model suggested that individuals without contact with pets or farm animals (median probability [89% HPD intervals] = 0.01 [0.00–0.03]) had a lower *Giardia* incidence compared to individuals in contact with pets (Figure 2; 0.07 [0.03–0.12]; difference contrast median [89% HPD intervals] = −2.44 [−4.58– −0.67]) or both pets and farm animals (0.06 [0.01–0.11]; contrast = −2.22 [−4.17 – −0.20]). Interestingly, age, gender, lifestyle, and travel history did not display correlations with variability in *Giardia* prevalence (Table 6).

**Table 6.** Outputs of a Bayesian generalized linear model with binomial distribution and logit link explaining a variation in *Giardia* occurrence in humans detected using qPCR method. HPD intervals not including zero suggest a statistical significance and a Bayes factor greater than 1 can be interpreted as evidence against the null, a Bayes factor greater than 3 can be considered as substantial evidence against the null.

Parameter	Median	HPD low	HPD high	Bayes factor
Intercept	−2.50	−4.05	−1.06	2.14
Age	0.00	−0.02	0.02	0.01
Sex [male]	−0.74	−1.77	0.22	0.74
Living [village]	−0.68	−1.90	0.46	0.54
Travel [no]	0.56	−0.67	1.72	0.50
Travel [out EU]	0.22	−0.76	1.23	0.26
Animal contact [no]	−2.30	−5.07	−0.08	5.12
Animal contact [pets]	0.26	−0.8	1.45	0.30

## Discussion

The intestinal protist *Giardia intestinalis* is considered an opportunistic pathogen with a cosmopolitan distribution. Higher prevalence rates, typically ranging from 20% to 64%, are frequently documented in low-income countries most likely attributed to low hygiene standards [24, 25]. In contrast, high-income countries report the presence of *Giardia* primarily in symptomatic cases [20].

To date, there exists a limited number of studies investigating the prevalence of *G. intestinalis* in gut-healthy individuals. Moreover, the majority of these studies have exclusively focused on children below the age of 16 [5, 8]. Therefore, the principal objective of our study was to address this knowledge gap by assessing the occurrence of *Giardia* in a diverse age group of gut-healthy individuals (0 to >60 years) within a high-income country, the Czech Republic. We employed qPCR as the main detection method.

Given that epidemiological studies on *G. intestinalis* encompass a variety of diagnostic methods to identify positive individuals, each with varying sensitivity and specificity, we opted to compare our data solely with studies that utilized conventional PCR (cPCR) and qPCR.

Contrary to expectations, our study revealed a *Giardia* prevalence of 7% in 296 gut-healthy individuals. However, this prevalence is notably lower when compared to the results of a few currently available studies focusing on asymptomatic subjects. For instance, recent surveys conducted in Spain reported asymptomatic *Giardia* prevalence of 17% and 18% [5, 8]. Conversely, low-income countries such as Brazil, Ethiopia, Argentina, and Mozambique exhibit considerably higher asymptomatic prevalence of *Giardia* ranging from 18% to 64% [6, 26–28]. The low prevalence observed in our study could be attributed to the broad age range of the subjects (0 to >60 years). It should be emphasized that the studies mentioned above were mainly conducted on asymptomatic children under 16 years of age. Hence, additional cross-sectional studies are needed to clarify the presence of *G. intestinalis* in the healthy population, employing qPCR as a diagnostic method, with particular focus on the adult population. Our results demonstrating the occurrence of *Giardia* in an asymptomatic population are consistent with the observations of Messa et al. [26], who found its higher incidence among asymptomatic individuals (32%) compared to individuals with diarrhea (20%).

For the accurate detection of intestinal protists and the assessment of their prevalence in gut-healthy individuals, the utilization of a precise and highly sensitive molecular method is imperative e.g. [29, 30]. This choice becomes particularly crucial in the case of *Giardia*, given the sensitivity and specificity challenges associated with existing molecular targeting protocols [18]. In our study, we initially conducted a specificity comparison for cPCR across three different genes [31–33], with the TPI gene protocol demonstrating the highest specificity [33].

Subsequently, we compared the sensitivity between TPI cPCR and qPCR for *Giardia* detection*.* This comparative analysis aimed to identify the most sensitive method, particularly in the context of our study focus on gut-healthy individuals with low colonization. Our findings corroborate the limited sensitivity of cPCR, especially for detecting weak *Giardia* colonizations (i.e. below 10^4^), as qPCR identified an additional 17 positive samples. Conventional coproscopical methods (e.g. Sheather flotations) only detected two positive samples.

In addition to assessing qPCR sensitivity, we quantified the intensity of *Giardia* colonization in positive samples by constructing a quantification curve. This curve was generated using a dilution series derived from a *Giardia* culture (range of 10^−1^–10^5^ cells *per* sample). We chose to use trophozoites from the culture instead of cysts to generate the quantification curve because we did not have *Giardia*-positive samples that contained enough cysts, even from experimental animals [34]. A major limitation of our study was the insufficient number of cysts available to construct the curve.

A surprising finding in our study is the remarkable sensitivity of qPCR in detecting of very low colonization intensities (from 10^−1^ cells *per* 1 qPCR reaction), the so-called fecal protist load (according to Šloufová *et al*. [29]). In contrast, cPCR only identified samples with moderate to high fecal *Giardia* load, especially those with more than 10^4^ cells. This contrast in sensitivity among different molecular protocols underscores the potential for biased information about the presence of *Giardia* in gut-healthy individuals when employing varying diagnostic approaches.

Our results indicate that asymptomatic cases are characterized by low fecal loads, typically falling within the range of 10^−1^–10^1^, which can only be detected by qPCR and remain undetectable by less sensitive methods. Additionally, our study underscores the difficulty in obtaining sequences for assemblage determination using cPCR, specifically for the TPI gene, in qPCR-positive samples with fecal *Giardia* loads below 10^3^. Consequently, the identification of assemblages was feasible in only seven out of 47 positive samples, all of which had fecal loads between 10^4^ and 10^6^. These identified assemblages included BIV in five rabbits, AII in one dog, and BIII in one human. Our findings align with those of Belkessa et al. [17], who also reported similar challenges related to the sensitivity of qPCR and the sequence obtaining via cPCR.

Another objective of our study was to investigate the presence of *Giardia* in animals closely associated with volunteers, focusing on possible zoonotic transmission. Our results revealed the *Giardia* positivity rate of 19% in these animals. However, there is a lack of comprehensive studies examining the presence of *Giardia* across a range of animal species by PCR. Nevertheless, our results appeared to be among the range of positivity rates reported in different animal species: 29% in dogs [35], 8% in cats [36], 4% in rabbits [37], 33% in pigs [38], 28% in cattle [39], 4% in guinea pigs [40], and 17% in horses [41].

Regarding the identification of assemblages, as mentioned above, we were able to confirm only five in rabbits and one in a dog based on an adequate colonization intensity for cPCR (> 10^4^). In one case, we detected *Giardia* by qPCR in both the owner and his pet (a rabbit). Unfortunately, we were unable to obtain a sequence from the owner's sample, primarily because of the low fecal *Giardia* load (10^1^). Consequently, zoonotic transmission could not be confirmed in this case.

The distribution of intestinal protists is influenced by various epidemiological factors such as living locality, contact with animals, travel history, age, and gender [21, 30]. Among these factors, only contact with animals significantly impacted *Giardia* occurrence in gut-healthy individuals in our study. Individuals in regular contact with animals, whether pets or farm animals, exhibited a higher prevalence, suggesting that close animal contact might play a pivotal role in zoonotic transmission [42, 43]. This aligns with a previous study in Brazil that identified shared sub-assemblages (BIV) between children and pet dogs, suggesting potential *Giardia* transmission [44]. However, our data cannot definitively confirm zoonotic transmission due to limited sequencing information as previously mentioned.

Interestingly, lifestyle factors, such as travel history and living locality, did not influence *Giardia* occurrence in the Czech gut-healthy individuals. While travel is often considered a significant predisposing factor [45, 46], our study did not reveal a higher prevalence among individuals who travelled within or outside of Europe compared to non-travellers. Additionally, rural residents, who are typically at a risk of exposure to potential sources of *Giardia* colonization [43, 47], did not show here a significantly higher *Giardia* prevalence compared to urban residents. This is consistent with a Colombian study reporting similar prevalence in both urban and rural areas [48].

*Giardia intestinalis* is most frequently found in children, particularly those under five years of age [43, 47]. A study by Muadica et al. [5] focused on healthy asymptomatic children aged 1–16 years, revealed a 17% prevalence (263/1512). Here, we did not observe any significant age-related effects. Regarding gender, we found no difference in occurrence between women (9%) and men (5%), which is consistent with a previous study [24].

## Conclusions

In conclusion, our study contributes significantly to filling the knowledge gap regarding the occurrence of *G. intestinalis* in gut-healthy individuals in a high-income region. However, we found a lower prevalence (7%) compared with previous studies, which may be due to the wider age range of the individuals included in our study. Our results highlight the importance of using accurate and highly sensitive molecular methods such as qPCR for precise *Giardia* detection. Traditional and conventional methods commonly employed in epidemiological studies often exhibit limited sensitivity and specificity.

Quantitative PCR has demonstrated exceptional sensitivity in detecting even weak *Giardia* colonization. However, the challenge of obtaining sequences by cPCR from qPCR-positive samples with low fecal loads remains an issue for future studies. In addition, our results highlight the need for further research using advanced tools to explore the genetic diversity of *Giardia* in animals and its potential transmission to humans.

In our study, close contact with animals was identified as a significant factor associated with a higher prevalence of *Giardia* in gut-healthy individuals, suggesting a possible role in zoonotic transmission. However, the ability to obtain sequences for assemblage identification is limited, preventing definitive confirmation in this regard.

## Supplementary Material

Supplemental Material

Supplemental Material

Supplemental Material

Supplemental Material
