# Supplementary material for: The opportunistic protist, Giardia intestinalis, occurs in gut-healthy humans in a high-income country
Source: Emerg Microbes Infect. 2023 Oct 10;12(2):2270077. doi: 10.1080/22221751.2023.2270077 (PMC10614719; doi:10.1080/22221751.2023.2270077)
Supplement: Supplemental Material [file TEMI_A_2270077_SM7039.docx]

**Supplementary Data**

**Supplementary Data 1:** Quantification curve for qPCR based on a decimal dilution series from the trophozoite culture.

Quantification curve was established to estimate fecal *Giardia* load using qPCR. The quantification curve was generated using trophozoite cultures (WB ATCC 30957, human isolate) ranging from of 10^-1^ to 10^5^ trophozoites per one qPCR reaction. The number of *Giardia* trophozoites was calculated using a Bürker’s chamber and then samples were serially diluted to appropriate aliquots, which were then subjected to DNA extraction.

**Supplementary Data 2:**

We encountered a problem with a separation in our data (no human individual without contact with an animal was positive), in which case a typical logistic regression would produce a biased estimate(s), often diverging to ± infinity. Bayesian computation method can effectively solve the problem using a weakly informative prior, e.g. with the normal distribution of mean=0 and SD=2. This prior has been set for all predictors and is generally preferred over the flat prior (Lemoine, 2019).^[[1]](#footnote-1)^ Using the package brms (version 2.18.0; Bürkner, 2019)^[[2]](#footnote-2)^ in R software (version 4.1.3; R Core Team, 2022),^[[3]](#footnote-3)^ we ran 4 × 10^3^ iterations with a burn-in phase of 1000 to obtain about 3000 effective samples per parameter for posterior inference. The Potential Scale Reduction Factor (R-hat) for all parameters was 1.00 suggesting a good convergence of the fit. For all posterior inferences, we used the package emmeans (version 1.8.4-1; Lenth, 2023).^[[4]](#footnote-4)^ We computed 89% highest posterior density (HPD) intervals, which are more stable than 95% intervals if the effective sample size for a parameter < 10 000 (Makowski *et* al. 2019).^[[5]](#footnote-5)^ However, re-calculating with 95% HPD intervals did not change the conclusions. Predictor effects were tested using HPD intervals, where intervals not overlapping zero suggested a significant difference. Additionally, we computed a Bayes factor (the Savage-Dickey density ratio) using the package bayestestR (version 0.13.0; Makovski *et* al. 2019)^5^ and assumed that values of 3 and higher suggest evidence for significant difference from the null value (e.g., Kruschke, 2018).^[[6]](#footnote-6)^

**Supplementary Data 3:**

We also tested the other two protocols for beta-giardin (BG) and small ribosomal subunit (SSU rRNA) to detect *G. intestinalis* but could not optimize them sufficiently under our laboratory conditions because of the lack of specificity and sensitivity of these protocols. We tested all three protocols on a trophozoite culture (TC) and a *Giardia*-positive animal fecal sample (B151). In the case of BG, we detected *Giardia* in TC but not in a positive *Giardia* sample (B151, fecal *Giardia* load 10^6^). Using the protocol for SSU, we were unable to detect *Giardia* in both the TC and the positive sample B151; although we saw a weak band in B151, we were not able to obtain *Giardia* sequences. Only the triosephosphate isomerase protocol (TPI) was specific enough to detect *Giardia* in both positive samples (TC and B151) and obtain sequences.

**Supplementary Data 4:** The gel image from a PCR dilution series generated from a culture of *G. intestinalis* trophozoites of known concentration (TC = trophozoite stock culture). We can also see weak bands for sample with 10^2^ trophozoites and 10^3^ trophozoites, however we were not able to obtain sequences of *Giardia* from these weak bands, only from the sample with 10^4^ trophozoites we obtained a *Giardia* sequence

**Supplementary Data 5:**

Table with data on *Giardia-*positive humans.

| **Sample** | **Gender** | **Age** | **City/village** | **Traveling Europe/outside Europe** | **Contact with animals** | **Results qPCR** | **Results cPCR** |
| --- | --- | --- | --- | --- | --- | --- | --- |
| B006/14 | M | 30 | city | Europe | pets only | positive | negative |
| B009/18 | M | 39 | city | both | pets only | positive | negative |
| B016/14 | F | 28 | city | Europe | pets only | positive | negative |
| B025/14 | F | 50 | city | no | pets only | positive | negative |
| B060/15 | F | 20 | city | Europe | both | positive | negative |
| B064/16 | F | 1,2 | village | no | pets only | positive | negative |
| B066/16 | F | 58 | city | no | pets only | positive | negative |
| B077/16 | F | 43 | village | no | both | positive | negative |
| B087/17 | M | 62 | city | both | pets only | positive | negative |
| B121/18 | M | 26 | village | Europe | pets only | positive | negative |
| B123/18 | F | 3 | city | both | pets only | positive | negative |
| B147/18 | F | 69 | city | no | both | positive | negative |
| B227/18 | F | 29 | city | Europe | pets only | positive | negative |
| B231/18 | F | 35 | city | both | pets only | positive | negative |
| B233/18 | F | 20 | city | Europe | pets only | positive | negative |
| B246/18 | F | 23 | city | both | pets only | positive | negative |
| B248/18 | F | 43 | city | both | pets only | positive | negative |
| B255/18 | F | 60 | village | Europe | both | positive | negative |
| B429/19 | M | 66 | city | both | both | positive | negative |
| B436/19 | F | 38 | city | Europe | pets only | positive | negative |
| B441/20 | F | 33 | city | both | pets only | positive | positive |
| B442/20 | M | 33 | city | both | pets only | positive | negative |

1. Lemoine NP. Moving beyond noninformative priors: why and how to choose weakly 2 informative priors in Bayesian analyses. Oikos, 2019. https://doi. org/101111/oik 05985. [↑](#footnote-ref-1)
2. Paul-Christian Bürkner. brms: An R Package for Bayesian Multilevel Models Using Stan. Journal of Statistical Software 2017; 80:1-28. doi:10.18637/jss.v080.i01. [↑](#footnote-ref-2)
3. R Core Team. R: A language and environment for statistical computing. R Foundation for Statistical Computing, Vienna, Austria, 2022. https://www.R-project.org/. [↑](#footnote-ref-3)
4. Russell V Lenth. emmeans: Estimated Marginal Means, aka Least-Squares Means. R package version 1.8.4-1, 2023. https://CRAN.R-project.org/package=emmeans. [↑](#footnote-ref-4)
5. Makowski D, Ben-Shachar M, Lüdecke D. bayestestR: Describing Effects and their Uncertainty, Existence and Significance within the Bayesian Framework. Journal of Open Source Software, 2019; 4:1541. doi:10.21105/joss.01541. [↑](#footnote-ref-5)
6. Kruschke JK (2018). Rejecting or accepting parameter values in Bayesian estimation. Advances in Methods and Practices in Psychological Science, 2018; 1:270– 280. https://doi.org/10.1177/2515245918771304. [↑](#footnote-ref-6)
