# Supplementary figures and images for: The opportunistic protist, Giardia intestinalis, occurs in gut-healthy humans in a high-income country
Source: Emerg Microbes Infect. 2023 Oct 10;12(2):2270077. doi: 10.1080/22221751.2023.2270077 (PMC10614719; doi:10.1080/22221751.2023.2270077)

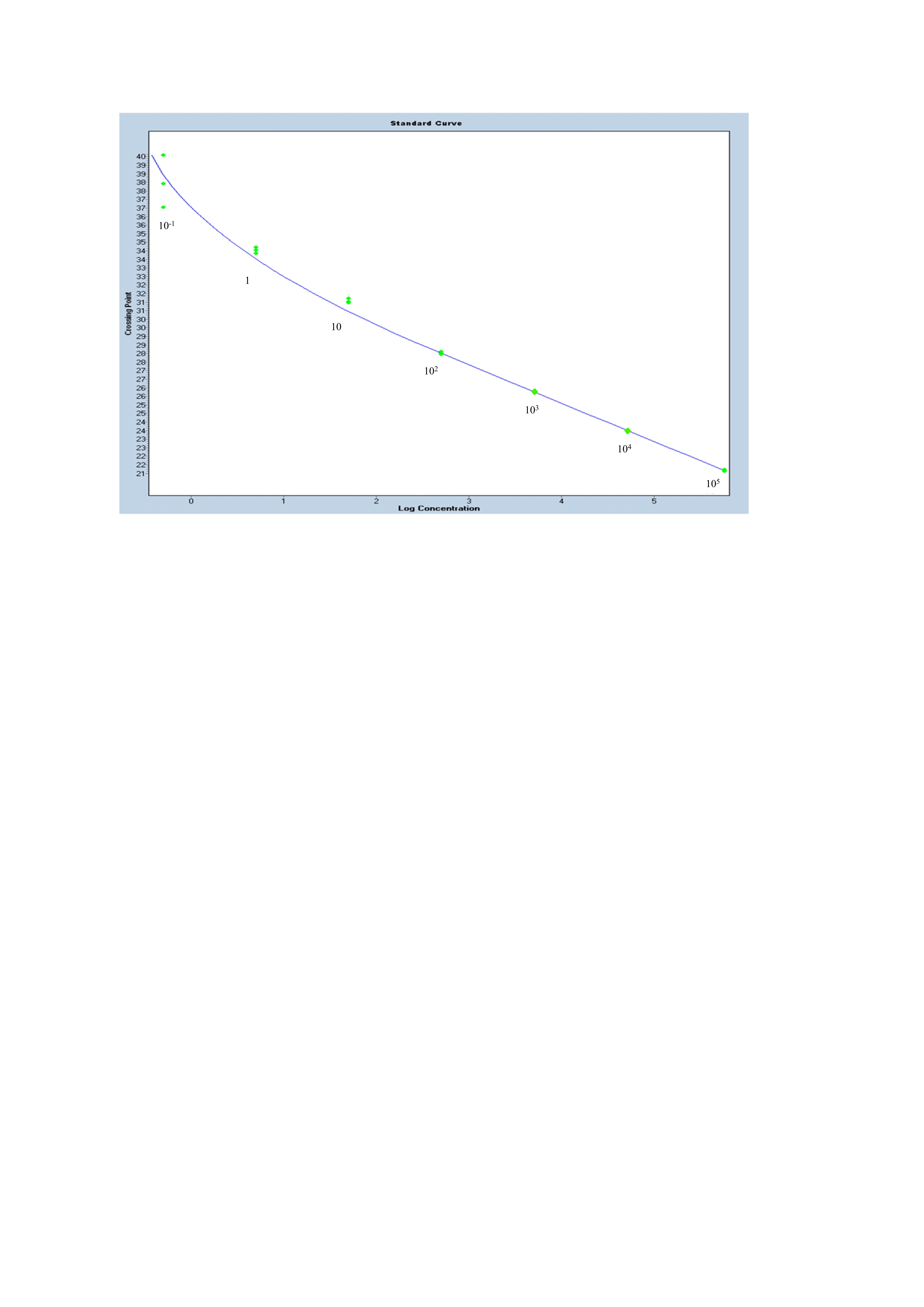

Supplement: Supplemental Material [file TEMI_A_2270077_SM7036.jpg]

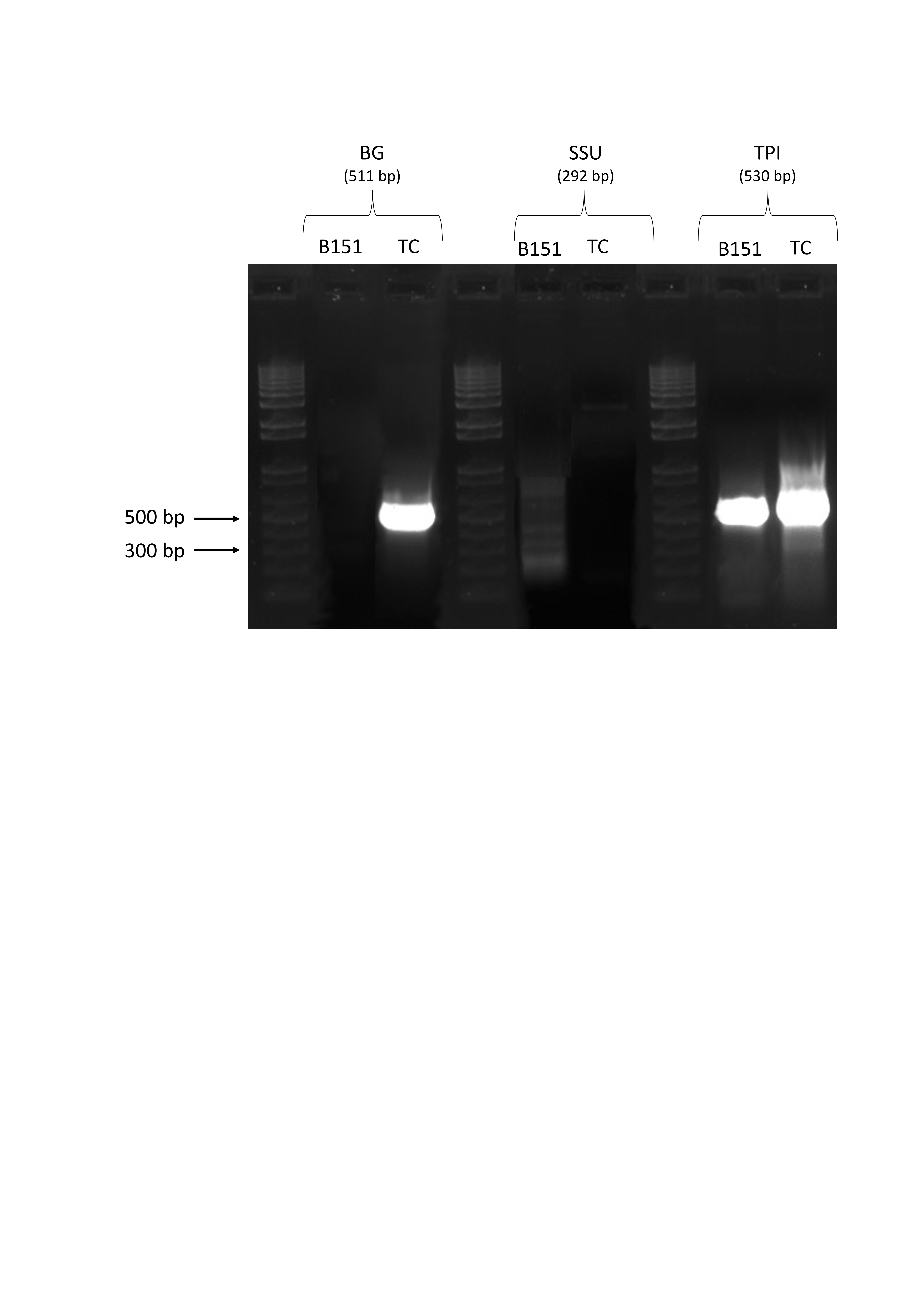

Supplement: Supplemental Material [file TEMI_A_2270077_SM7033.jpg]

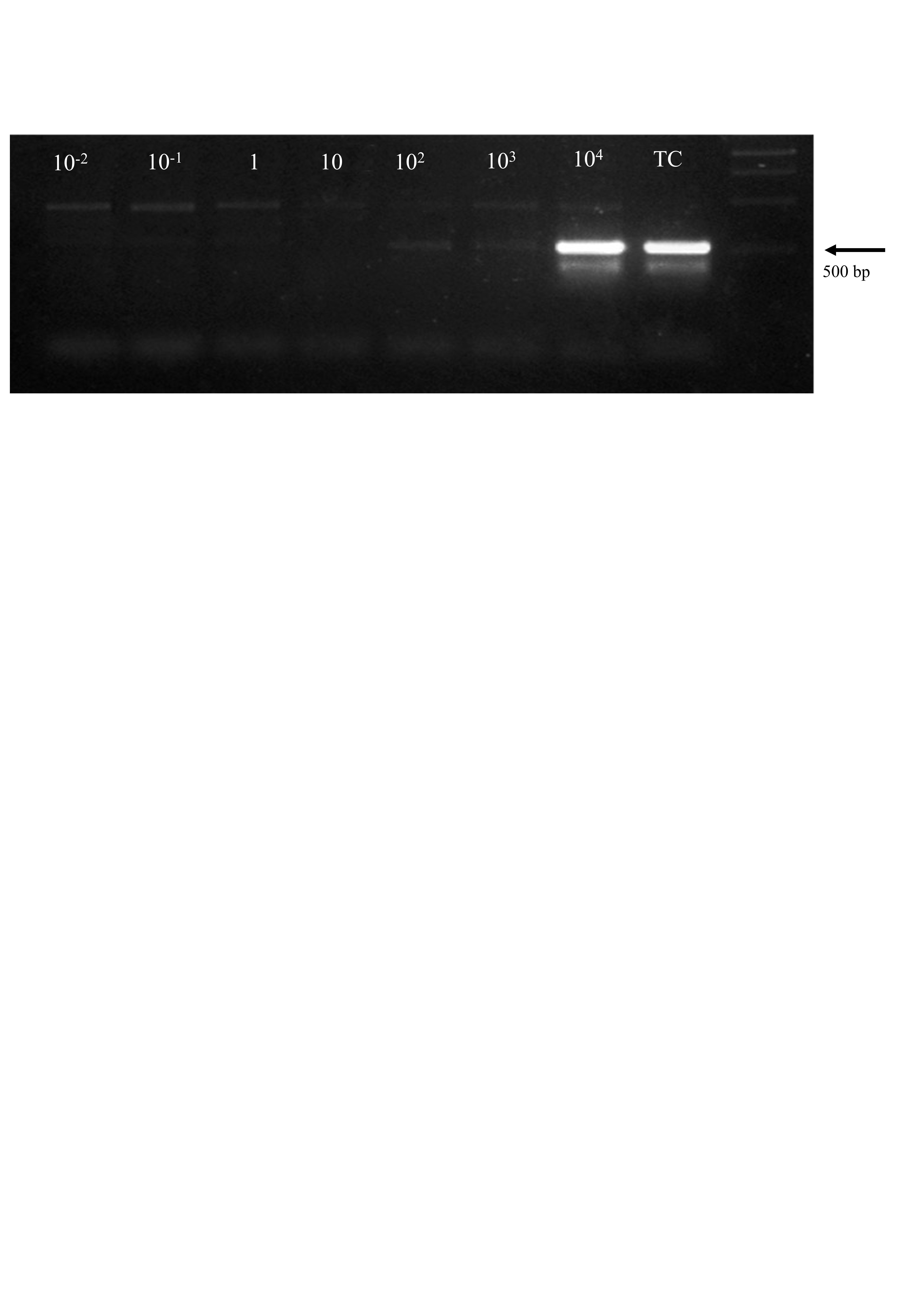

Supplement: Supplemental Material [file TEMI_A_2270077_SM7028.jpg]
